# Short-range UV-LED irradiation in postmenopausal osteoporosis using ovariectomized mice

**DOI:** 10.1038/s41598-021-86730-0

**Published:** 2021-04-12

**Authors:** Satoshi Ochiai, Yoshihiro Nishida, Yoshitoshi Higuchi, Daigo Morita, Kazuya Makida, Taisuke Seki, Kunihiro Ikuta, Shiro Imagama

**Affiliations:** 1 Department of Orthopaedic Surgery, Nagoya University Graduate School of Medicine, Nagoya, Japan; 2 Department of Rehabilitation Medicine, Nagoya University Hospital, 65 Tsurumai-cho, Showa-ku, Nagoya, 466-8550 Japan; 3 Medical Genome Center, Nagoya University Hospital, Nagoya, Japan

**Keywords:** Endocrinology, Health care

## Abstract

Postmenopausal osteoporosis is crucial condition that reduces the QOL of affected patients just like aged type osteoporosis. The aim of this study was to evaluate the effectiveness of short-range UV-LED irradiation in postmenopausal osteoporosis using ovariectomized mice. Preliminary experiments identified the time of onset of osteoporosis after ovariectomy (8 weeks) in our model. We have set up a total of 4 groups (n = 8/group); vitamin D-repletion with UV irradiation (Vit.D+UV+), vitamin D-repletion without UV irradiation (Vit.D+UV−), vitamin D-deficiency with UV irradiation (Vit.D-UV+), vitamin D-deficiency without UV irradiation (Vit.D-UV−), and. From 8 weeks after ovariectomy, UV was irradiated for 24 weeks. At the time of 16 and 24 weeks’ irradiation, serum Vit.D levels, various markers of bone metabolism, bone mineral density, and bone strength were evaluated, and histological analyses were performed. In addition, muscle strength was analyzed. Serum 25-hydroxyvitamin D [25 (OH) D] levels at 40 and 48 weeks of age were increased in the Vit.D-UV+ group compared to the Vit.D-UV−group. Cortical thickness evaluated with micro-CT and strength of bone were significantly higher in Vit.D-UV+ group than those in Vit.D-UV− group. There was no difference in muscle strength between Vit.D-UV+ group and Vit.D-UV− group. No obvious adverse effects were observed in UV-irradiated mice including skin findings. Short-range UV irradiation may ameliorate postmenopausal osteoporosis associated with a state of vitamin D deficiency.

## Introduction

Osteoporosis and associated fractures are associated with poor quality of life, increased risk of physical harm, and significant financial burden^1^. The incidence of osteoporotic hip fractures in American women is estimated to be approximately 230,000 per year^2^. If a novel device that was efficient, cheap, and minimally invasive to the body could be developed, the healthy life expectancy could be extended by preventing the bedridden state, and medical costs could be reduced by reducing the number of patients needing surgery. Vitamin D is a molecule that plays a central role in bone metabolism, and 90% of the body's vitamin D is produced by the skin upon exposure to sunlight and ultraviolet rays^3^. Despite this importance of vitamin D, the majority of older women with osteoporosis are reported to be deficient in vitamin D, possibly because of reduced opportunities to go out, and subsequent reduced sunbathing^4^. In addition to the reduction of sunbathing, insufficient intake of vitamin D from the diet has become a global problem^5^. There are also reports that vitamin D is deficient not only in the elderly but also in postmenopausal women^6–8^. Against this background, the Japan Osteoporosis Society recommends increased vitamin D intake and exercise to prevent fractures^9–11^. Up to now, no medical device for treating osteoporosis has been developed. The development of a medical device that effectively produces vitamin D would be a revolutionary treatment for osteoporosis for not only elderly patients with osteoporosis, but also postmenopausal osteoporosis ones. For the purpose of prevention and treatment of osteoporosis, we aim to develop a therapeutic device that efficiently produces vitamin D in the body using the light emitting diode (LED) technology. Our previous studies revealed that UVA (316 nm), UVB (305 nm, 290 nm, 282 nm) and UVC (268 nm) wavelengths all had sufficient vitamin D production effects in C57BL/6 mice^12^. In addition, in a senile mice model that mimics elderly osteoporosis (SAMP6) with vitamin D deficiency, it was confirmed that irradiation with UV-LED (305 nm) increased vitamin D production, bone density and strength^13^. The irradiation conditions used in those studies of mice were considered to be harmful to humans. Therefore, we then determined the minimum irradiance and dose of short-range UV-LED that effectively increases serum levels of vitamin D in a senile model of SAMP6^14^. The determined condition also increased the bone mineral density and strength. The next remaining issue is to verify the effects of short-range LED devices on the effective increase of vitamin D and subsequent bone strength in the postmenopausal type of osteoporosis.

In this study, we investigated whether irradiation of short-range UV-LED supplies sufficient levels of serum Vitamin D, and improves osteoporosis in a mice model with postmenopausal osteoporosis.

## Results

### Preliminary experiments

**Figure 1:**
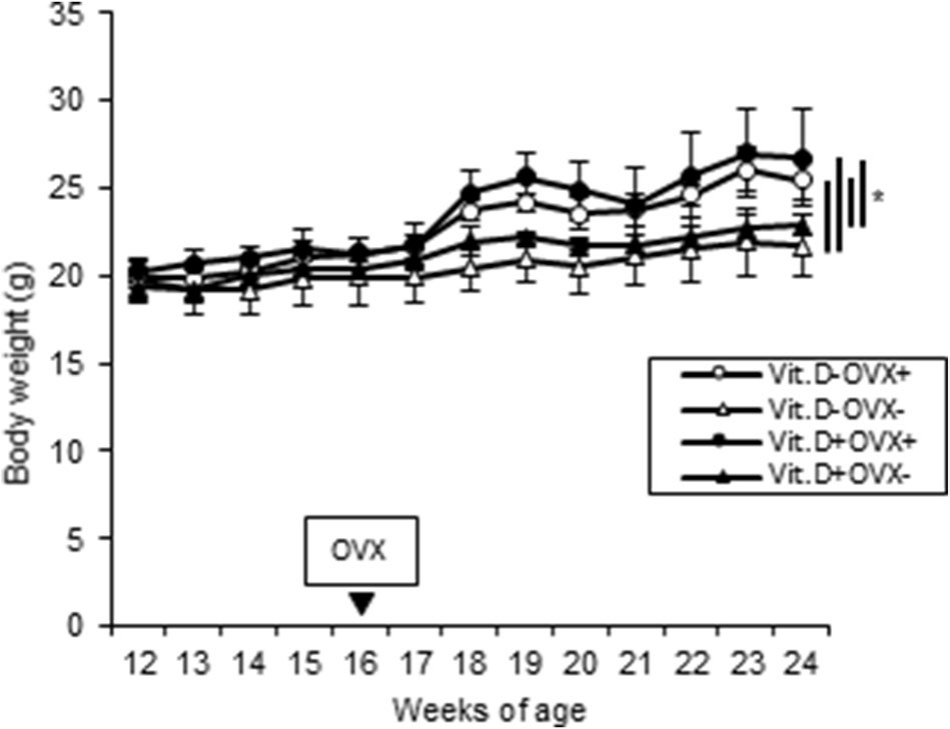
Body weight growth curves of ovariectomized (OVX +) and sham-operated (OVX−) female mice. Both groups were fed either a vitamin D–containing diet (Vit.D +) or diet without vitamin D (Vit.D−). Sham-operation or ovariectomy was performed at 16 weeks of age. *p < 0.05. OVX, ovariectomy.

**Figure 2:**
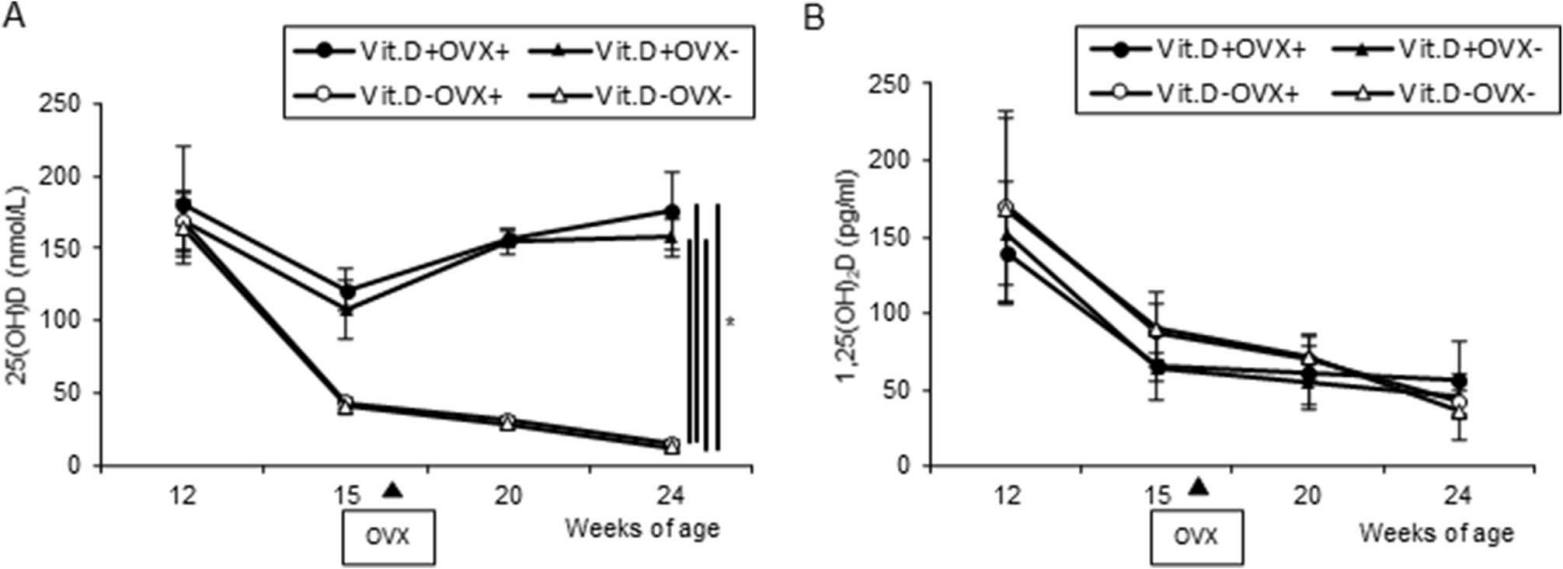
Serum levels of 25(OH)D and 1,25(OH)2D in preliminary study. Sera were collected at 12 weeks of age (initiation of diet with vitamin D–deficient or vitamin D–containing), 15 weeks, and 20, 24 weeks, and subjected to 25(OH)D and 1,25(OH)2D examination. (**A**) Serum levels of 25(OH)D of preliminary experiment. (**B**) Serum levels of 1,25(OH)2D of preliminary experiment. *p < 0.05.

**Figure 3:**
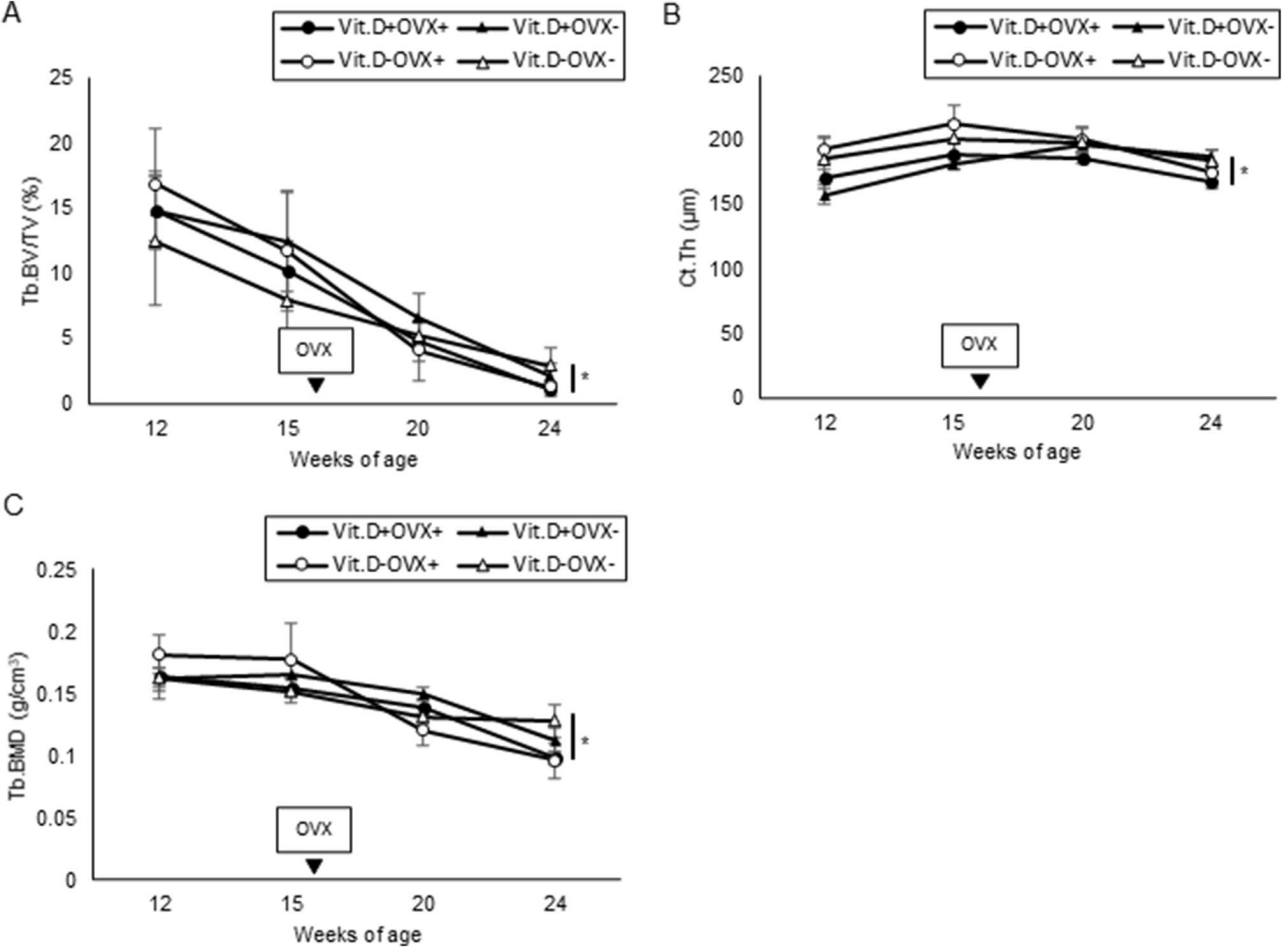
Bone morphological parameters by micro-CT analyses in preliminary study. Each parameter was measured using micro-CT at 12 weeks of age (initiation of diet with vitamin D–deficient or vitamin D–containing), 15 weeks, and 20, 24 weeks. (**A**) Trabecular percent bone volume [Tb.BV/TV] (**B**) Cortical thickness [Ct.Th] (**C**) Trabecular bone mineral density [Tb.BMD] *p < 0.05. CT, computed tomography.

As the purposes of the preliminary experiment, we wanted to evaluate whether ovariectomy (OVX) was appropriately performed to create a postmenopausal osteoporosis model. In addition, we examined how many weeks after OVX bone changes occur in C57BL/6 female mice, which provided important basic data for determining the irradiation timing of the main experiment. As shown in Fig. 1, OVX+ mice gradually gained more weight than OVX-mice. Regardless of Vit.D+ and Vit.D− status, OVX+ mice showed significant weight gain (*p* < 0.05) compared with OVX- mice at 24 weeks of age. Neither 25-hydroxyvitamin D [25 (OH) D] nor 1,25-dihydroxyvitamin D [1,25 (OH)_2_D] was affected by OVX (Fig. 2). As expected, Vit.D− mice showed significantly lower 25(OH)D levels compared to those of Vit.D+ mice (*p* < 0.05) (Fig. 2A), while there were no significant differences in serum levels of 1,25(OH)D between the groups (Fig. 2B). On Micro-CT, Trabecular percent bone volume (Tb.BV/TV), cortical thickness (Ct.Th), and bone mineral density (BMD) all showed a significant decrease 8 weeks after OVX (Fig. 3). All parameters were significantly lower in the Vit.D− OVX+ group than Vit.D− OVX- group (*p* < 0.05).

### Main experiments

#### Body weight and side effects

**Figure 4:**
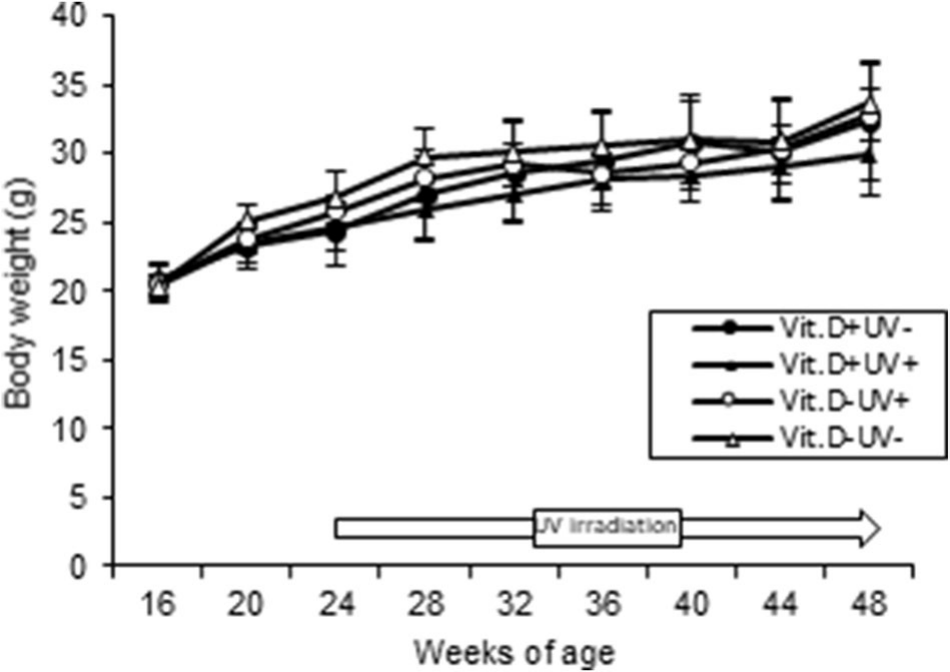
Body weight growth curves of ovariectomized female mice. Ovariectomized mice were divided into 4 groups, and fed either vitamin D–containing diet (Vit.D +) (2 groups) or diet without vitamin D (Vit.D−) (2 groups) from OVX weeks of age. UV was either irradiated or not from 24 to 48 weeks of age, the end of the study. The mice were finally divided into 4 groups. *UV* ultraviolet irradiation.

All mice in the main experiments were ovariectomized at 16 weeks of age. Gains of body weight tended to be lower in the Vit.D+UV+ group but not significantly so (Fig. 4). During the irradiation period, no apparent complications were observed in the irradiated mice macroscopically including skin erythema.

#### Serum metabolites

**Figure 5:**
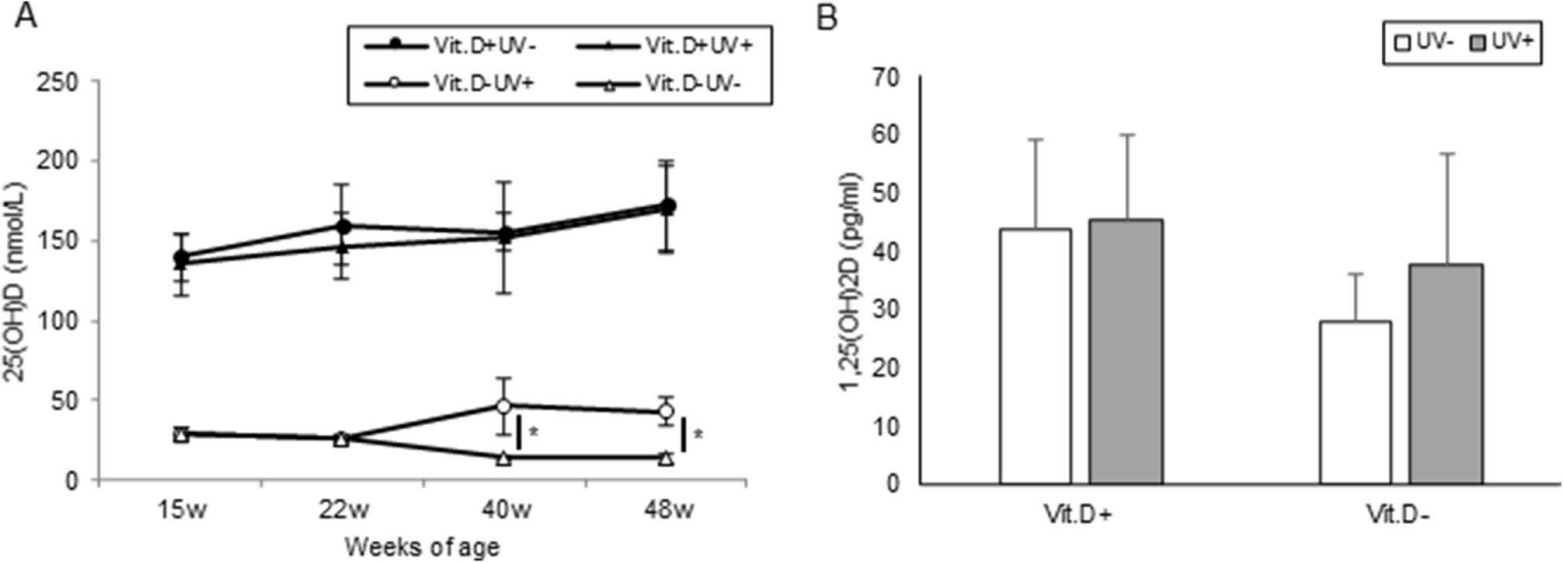
Serum levels of 25(OH)D and 1,25(OH)2D in main study. Sera for 25(OH)D examination were collected at 15 weeks of age, 22 weeks, 40 weeks (16-weeks of UV irradiation), 48 weeks (24-weeks of UV irradiation). Sera for 1,25(OH)2D examination were collected at 48 weeks (24-weeks of UV irradiation). (**A**) Serum levels of 25(OH)D. (**B**) Serum levels of 1,25(OH)2D. *p < 0.05. *Vit.D*− vitamin D-deficient diet; *Vit.D*+ vitamin D-replete diet; *UV* ultraviolet irradiation.

**Figure 6:**
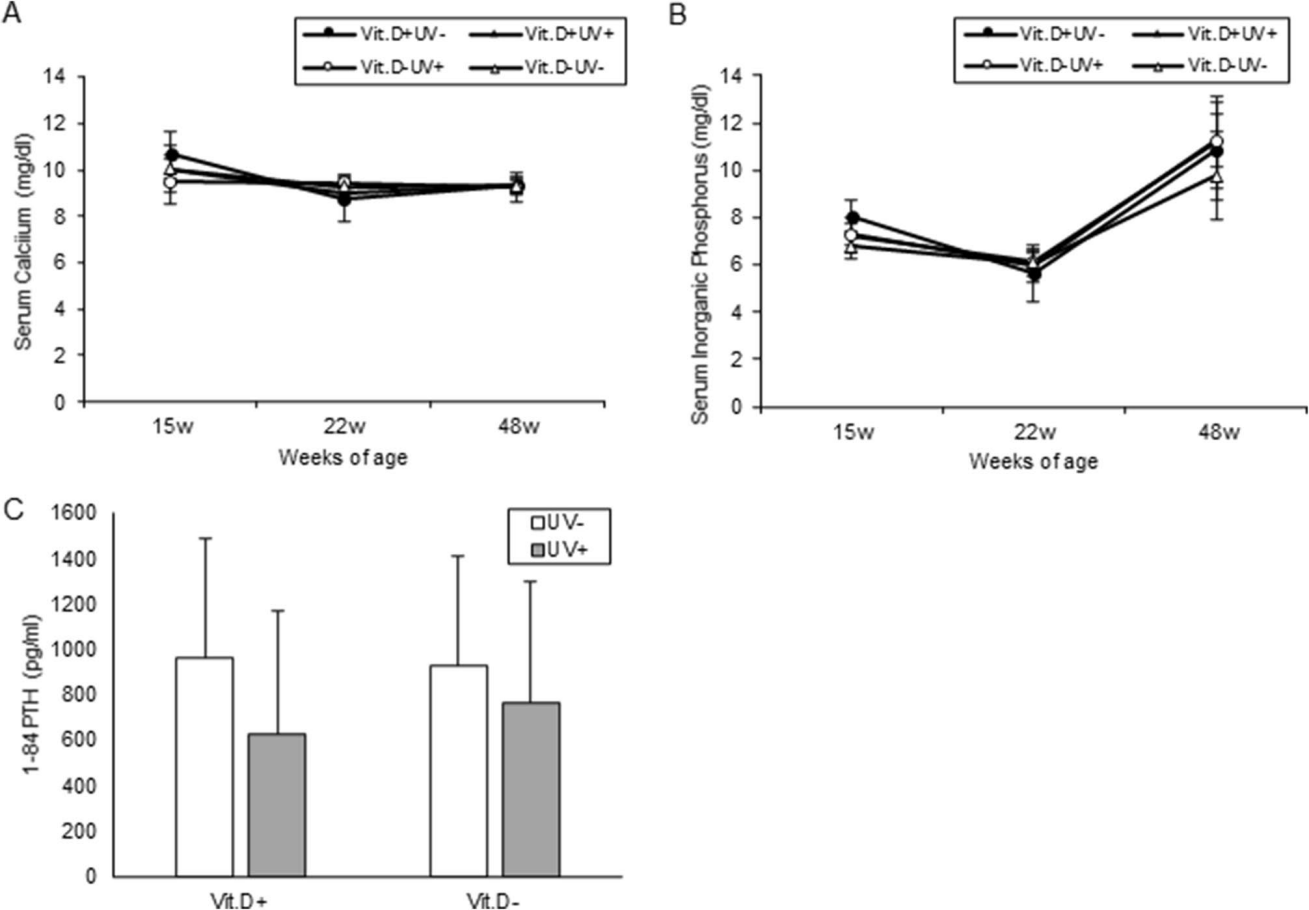
Serum levels of calcium, inorganic phosphorus, and 1–84 PTH. Sera for calcium and inorganic phosphorus determination were collected at 15 weeks of age, 22 weeks, and 48 weeks (24-weeks of UV irradiation). Sera for 1–84 PTH determination were collected at 48 weeks (24-weeks of UV irradiation). (**A**) Calcium. (**B**) Inorganic phosphorus. (**C**) 1–84 PTH. *PTH* parathyroid hormone; *Vit.D*− vitamin D-deficient diet; *Vit.D*+ vitamin D-replete diet; *UV* ultraviolet irradiation.

In the Vit.D+ group, no significant difference was observed in the serum 25 (OH) D value between the irradiated group and non-irradiated group. Whereas, in the Vit.D− group, a significant increase (*p* < 0.05) in serum 25 (OH) D was observed in the irradiation group at 40 and 48 weeks of age (Fig. 5A). There was no significant difference in serum 1.25 (OH)_2_D at 48 weeks of age (Fig. 5B). No differences were observed in serum levels of Ca or IP among the four groups (Fig. 6A,B, respectively). Although it was not statistically significant, serum levels of PTH in the UV irradiation groups were lower than those in the non-irradiated groups (Fig. 6C).

#### Analyses using micro-computed tomography (CT)

**Figure 7:**
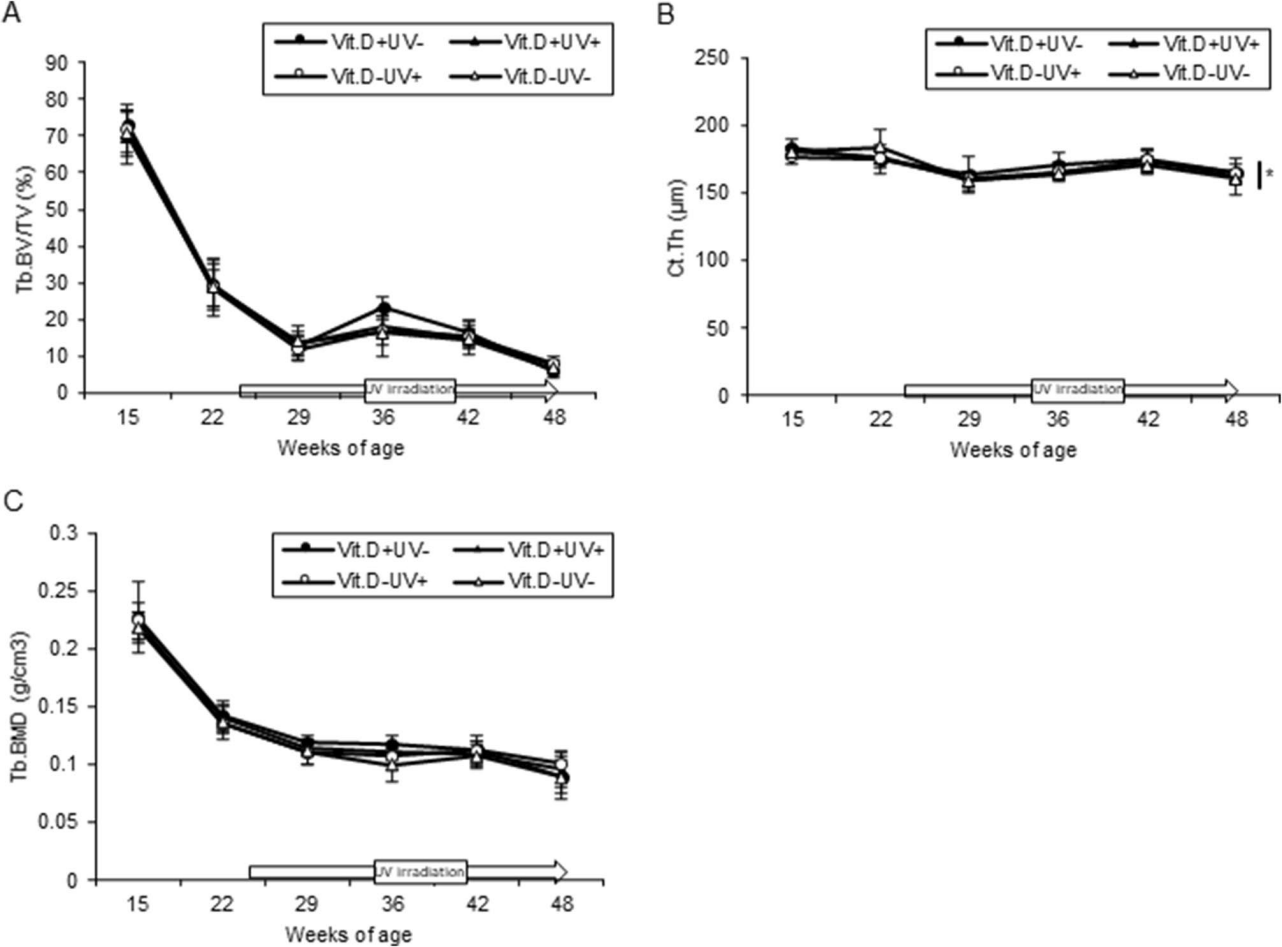
Bone morphological parameters with micro-CT analyses in main study. Each parameter evaluated by micro-CT is compared at 15, 22, 29, 36, 42, 48 weeks of age. (**A**) Trabecular percent bone volume [Tb.BV/TV] (**B**) Cortical thickness [Ct.Th] (**C**) Trabecular bone mineral density [Tb.BMD] *p < 0.05. *CT* computed tomography; *Vit.D*−, vitamin D-deficient diet; *Vit.D*+ , vitamin D-replete diet; *UV* ultraviolet irradiation.

In the micro-CT of the femur, there was no significant difference in bone mineral density or bone morphology between the irradiated and non-irradiated groups in the Vit.D+ group. On the other hand, in the Vit.D− group, all parameters increased in the irradiated group (Table 1). Among the parameters, cortical thickness of the metaphysis was significantly increased in the irradiated group compared to the non-irradiated group after 24 weeks of irradiation (Fig. 7B, Table1). On the other hand, there was no significant difference in Tb.BV/TV and Tb.BMD among the four groups (Fig. 7A,C, respectively).

**Table 1:** Bone morphology with micro-CT measurement at 48 weeks of age.

	Vit.D+		Vit.D−	
	UV−	UV+	UV−	UV+
Tb.BV/TV (%)	6.0 ± 1.8	7.4 ± 1.3	6.6 ± 1.2	8.1 ± 1.9
Tb.Th (µm)	58.9 ± 10.4	57.9 ± 5.15	62.6 ± 8.94	65.1 ± 10.0
Tb.N (1/mm)	1.03 ± 0.32	1.27 ± 0.16	1.13 ± 0.16	1.17 ± 0.19
Tb.Sp (µm)	813 ± 59	754 ± 89	712 ± 58	800 ± 68
Tb.BMD (mg/cm^3^)	88 ± 17	95 ± 14	89 ± 13	101 ± 9.5
Ct.Th (µm)	165 ± 5.7	162 ± 12	160 ± 1.9*	164 ± 2.8

*Significant difference compared with Vit.D− UV+ group, *p* < 0.05.

*CT* computed tomography; *Vit.D*− , vitamin D-deficient diet; *Vit.D*+ , vitamin D-replete diet; *UV* ultraviolet irradiation; *BV* bone volume; *TV* tissue volume; *Tb.BV/TV* trabecular percent bone volume; *Tb.Th* trabecular thickness; *Tb.N* trabecular number; *Tb.Sp* trabecular separation; *Tb.BMD* trabecular bone mineral density; *Ct.Th* cortical thickness.

#### Mechanical test

**Figure 8:**
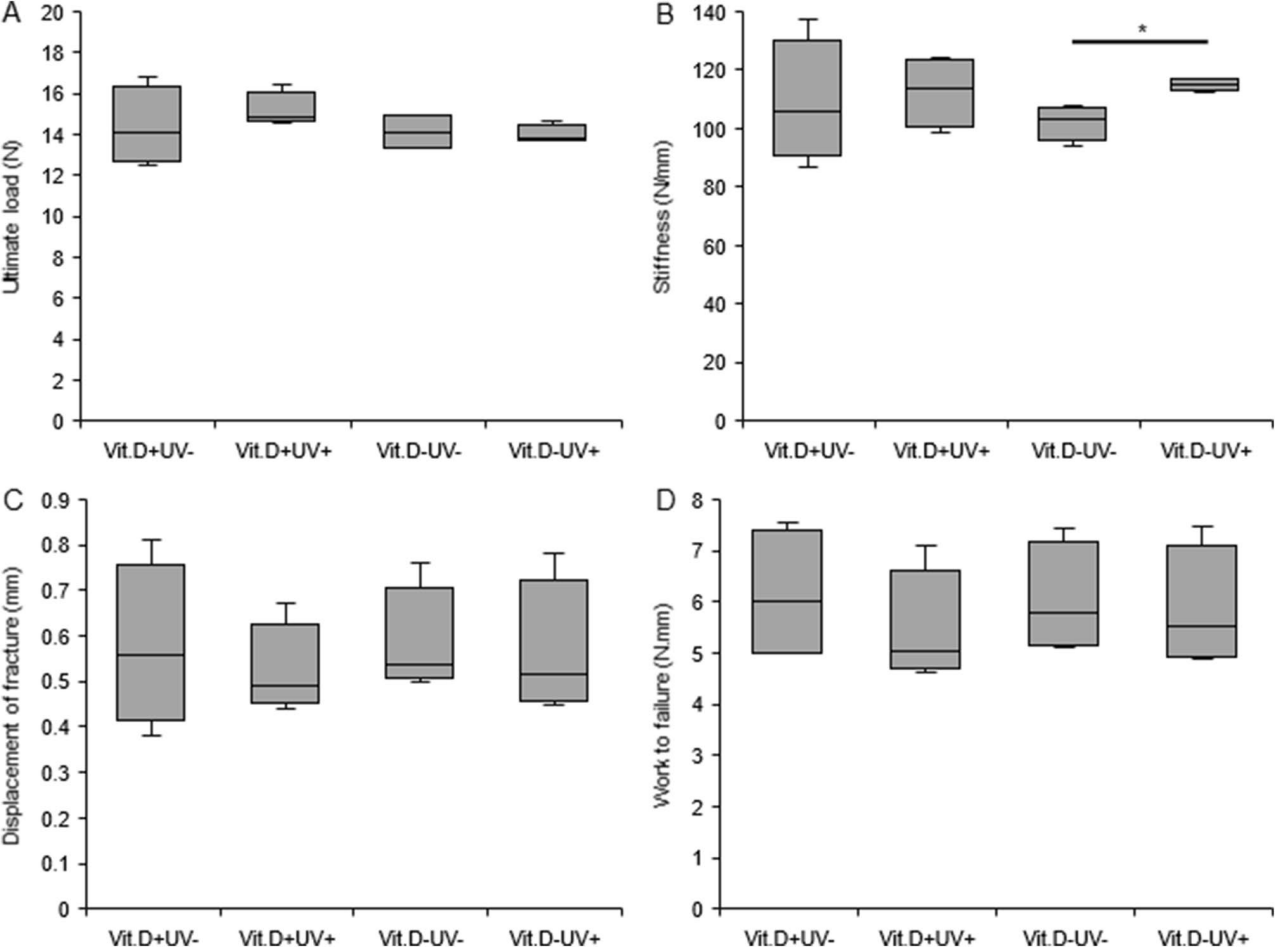
Results of mechanical test. Three point bending test of femur was performed at 48 weeks of age, and plotted. (**A**) Ultimate load (**B**) Stiffness (**C**) Displacement of fracture (**D**) Work to failure *p < 0.05. *Vit.D*− vitamin D-deficient diet; *Vit.D*+ vitamin D-replete diet; *UV* ultraviolet irradiation; *N* newton.

In the bone strength test of femur using the three-point bending test, there was no significant difference between the irradiated and non-irradiated groups in the Vit.D+ group. On the other hand, in the Vit.D− group, the stiffness of the irradiated group was significantly increased as compared with the non-irradiated group (*p* < 0.05) (Fig. 8). There was no significant difference in other parameters.

#### Real-time RT-PCR analysis

**Figure 9:**
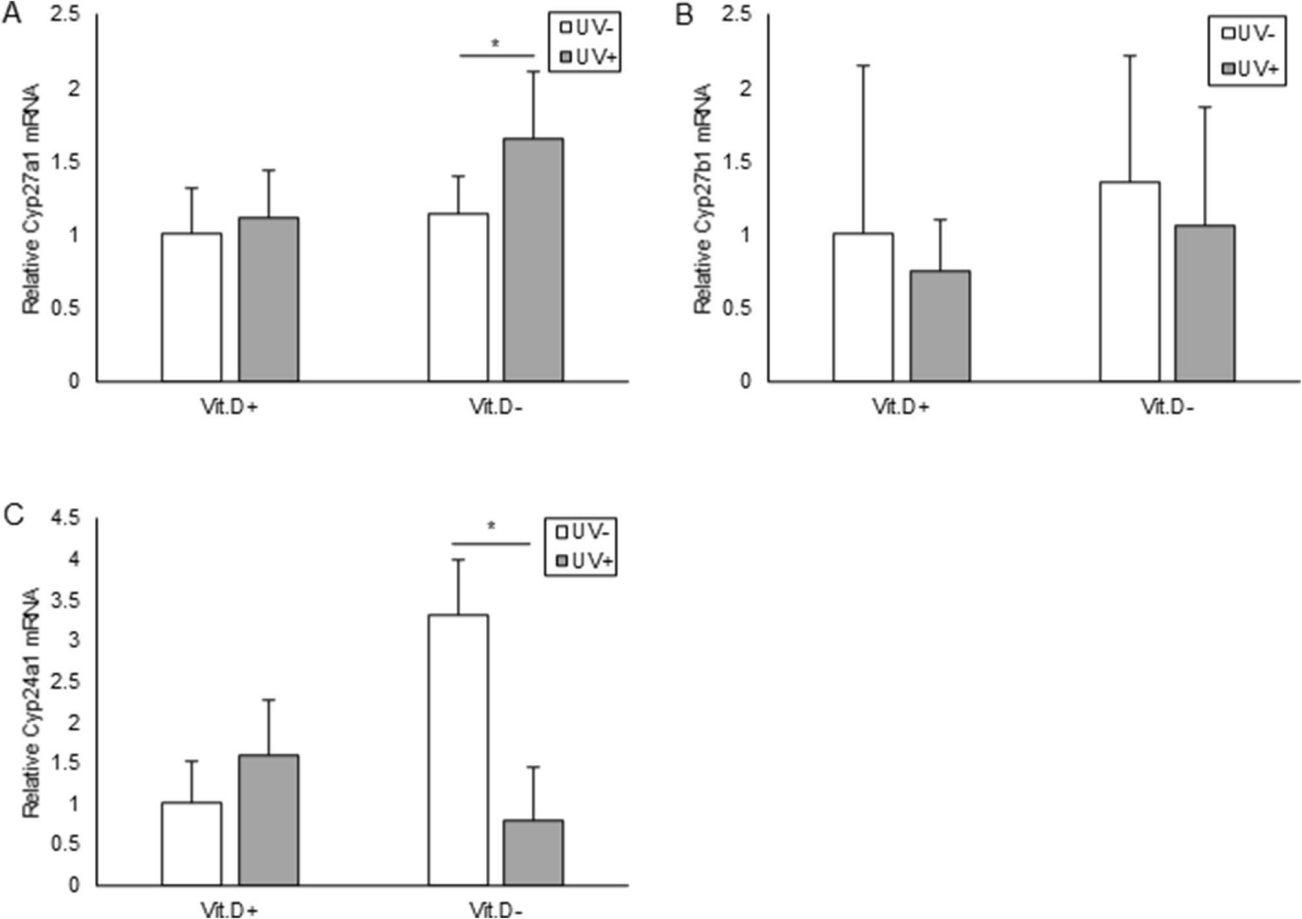
Expression levels of mRNA correlated with vitamin D metabolism. Relative expression levels of target mRNA are graphed with reference to that in Vit.D+ UV− group defined as 1.0. Levels of target mRNA were normalized with those of Gapdh mRNA, and expressed. (**A**) Relative expression levels of Cyp27a1 mRNAs. (**B**) Relative expression levels of Cyp27b1 mRNAs. (**C**) Relative expression levels of Cyp24a1 mRNAs. *p < 0.05. *Vit.D*− vitamin D-deficient diet; *Vit.D*+ vitamin D-replete diet; *UV* ultraviolet irradiation.

**Figure 10:**
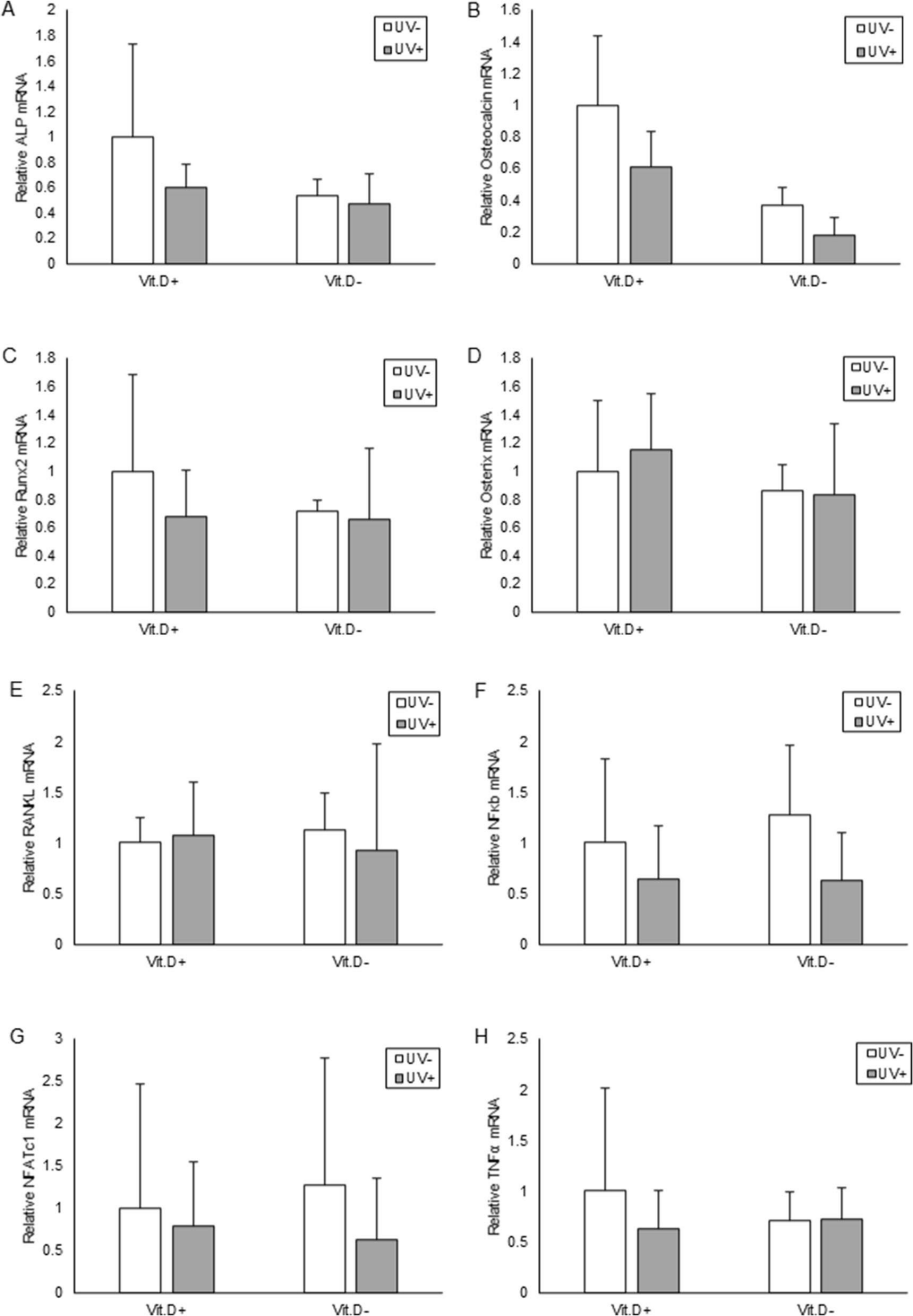
Expression levels of mRNA correlated with bone metabolism. Relative expression levels of target mRNA are graphed with reference to that in Vit.D+ UV− group as defined 1.0. Expression levels of target mRNA were normalized with those of Gapdh mRNA. (**A**) Relative ALP mRNAs. (**B**) Relative Osteocalcin mRNAs. (**C**) Relative Runx2 mRNAs. (**D**) Relative expression levels of Osterix mRNAs. (**E**) Relative RANKL mRNAs. (**F**) Relative expression levels of NFκb mRNAs. (**G**) Relative expression levels of NFATc1 mRNAs. (**H**) Relative expression levels of TNFα mRNAs. *p < 0.05. *Vit.D*− vitamin D-deficient diet; *Vit.D*+ vitamin D-replete diet; *UV* ultraviolet irradiation; *ALP* alkaline phosphatase; *Runx2* Runt-related transcription factor 2; *RANKL* receptor activator of NFκB ligand; *NFκB* nuclear factor kappa B; *NFATc1* nuclear factor of activated T cells.

Expression levels of mRNA was investigated for C25-hydroxylases (*Cyp27a1*), 25 hydroxyvitamin D-1-alpha hydroxylase (*Cyp27b1*), and 1,25-dihydroxyvitamin D 24-hydroxylase (*Cyp24a1*) with real-time RT-PCR. The mRNA levels of *Cyp27a1*, a responsible enzyme for the conversion of vitamin D into the stored form, 25(OH)D, increased in UV+ group compared to those in UV− group of both Vit.D+ and Vit.D− groups. In Vit.D− groups, the mRNA levels of *Cyp27a1* in UV+ group were significantly higher than those in UV− group (*p* = 0.048) (Fig. 9A). There were no significant differences in mRNA levels of *Cyp27b1*, a responsible enzyme for the conversion of 25(OH)D into the active form 1,25(OH)_2_D in the kidney (Fig. 9B). In Vit.D− groups, the mRNA levels of *Cyp24a1*, a responsible enzyme for the conversion of active 1,25(OH)_2_D into the inactive form, was significantly lower in UV+ group than in UV− group (*p* = 0.0012) (Fig. 9C). We also investigated levels of mRNA expression for molecules related to bone metabolism; *ALP*, *Osteocalcin*, *Runx2*, *Osterix* as bone formation related genes; *RANKL*, *NFκb*, *NFATc1*, *TNFα* as bone resorption related genes. Using tibia specimens at 48 weeks of age, there were no significant differences in expressions of any of the molecules between groups that had received UV irradiation or not (Fig. 10).

#### Bone histology

**Figure 11:**
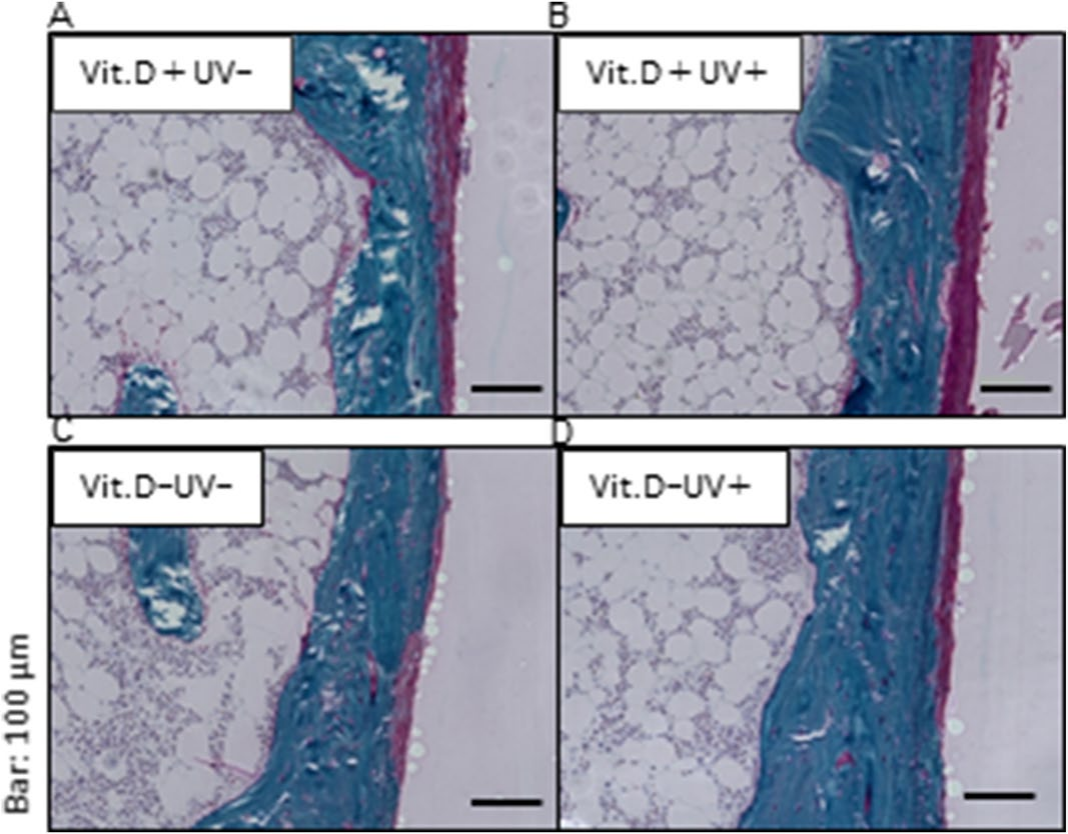
Villanueva Goldner staining. Stained coronal sections were presented of the medial metaphysis of right femurs at 48 weeks of age (original magnification × 200, bars indicate 100 μm). (**A**) Vit.D+ UV− group. (**B**) Vit.D+ UV+ group. (**C**) Vit.D− UV− group. (**D**) Vit.D− UV+ group. *Vit.D*− vitamin D-deficient diet; *Vit.D*+ vitamin D-replete diet; *UV* ultraviolet irradiation.

On Villanueva Goldner staining, cortical thickness was thicker in Vit.D-UV+ mice than in VitD-UV− ones, which correlated with the results of the CT examination (Fig. 7B, Table 1). Regarding red-colored osteoid tissues representing incomplete calcification of the bone matrix, a small amount was observed in all four groups, with no apparent difference among them (Fig. 11).

#### Grip strength

**Figure 12:**
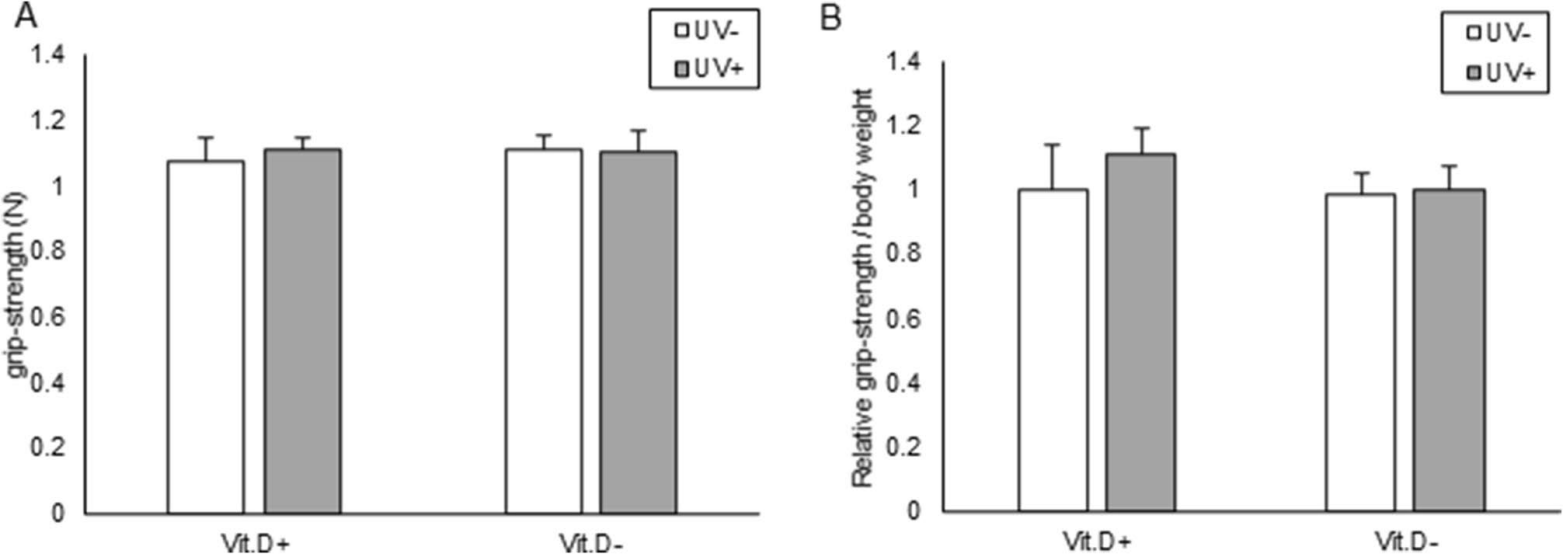
Muscle strength analyses. (**A**) Grip strength at 48 weeks of age was determined with reference to that in Vit.D+ UV− group as 1.0. measured in 15 trials per mouse. (**B**) Grip strength normalized with body weight. *Vit.D*− vitamin D-deficient diet; *Vit.D*+ vitamin D-replete diet; *UV* ultraviolet irradiation.

As indicated in Fig. 12, no significant difference was observed in grip strength evaluated at 48 weeks of age among the four groups, even if divided by body weight.

## Discussion

In our previous report, we confirmed the efficacy of short-range UV irradiation using an LED device on serum Vit.D, bone density and strength, and histological change of bone, in addition to muscle mass and strength focusing on low metabolic rotation type osteoporosis using an aged mice model of SAMP6. The results of the study also demonstrated that adverse effects, particularly on the skin, did not occur using the appropriate illuminance and dose determined by an effective minimum energy experiment^14^. Because postmenopausal osteoporosis is a social problem like in the elderly type, the next task was to investigate the effectiveness of UV irradiation with the LED device for hypermetabolizing osteoporosis. This is the first report to examine the effectiveness of UV irradiation with an LED device using OVX mouse, which is a model of postmenopausal osteoporosis.

Prior to the start of the main study, a preliminary requisite is a confirmation of whether the ovariectomized mice used in the present experiment are suitable as postmenopausal osteoporosis mice. Eight weeks after ovariectomy, significant decreases of Tb, BV/TV, Ct. Th, Tb, and BMD were observed, consistent with the results of the previous report using the same mice strain, C57BL female mice. In this report, a significant decrease of BMD was observed 6 weeks after ovariectomy^15^. We also set up a Vit.D+ group and a Vit.D− group in ovariectomized mice. This is because postmenopausal osteoporosis involves not only estrogen deficiency but also other factors including the condition of Ca and vitamin D deficiency^16^.

The results of the present study demonstrated intriguingly that no significant benefits of UV irradiation on serum Vit. D value, bone morphology or strength could be observed in Vit.D+ group, whereas all of these parameters increased significantly with UV-LED in Vit.D− group. A previous study have compared phototherapy with vitamin D supplies in humans, suggesting that phototherapy increased 25 (OH) D levels more than vitamin D supplies^17^. However, since it irradiates the UV-B, it cannot be compared with the present study. As previous studies reported, UV irradiation has photodegradation effects on Vit. D, there is a possibility that photodegradation of Vit. D by UV irradiation might cause the no difference in Vit. D+ group^18,19^. Theoretically, an efficient increase of serum Vit. D could be expected with a short-range LED device, followed by increases of BMD and bone strength particularly in postmenopausal woman under the condition of Vit. D deficiency. More than 75 million people worldwide suffer from osteoporosis, 80% of whom are postmenopausal women, and there is a direct link between estrogen deficiency and the development of osteoporosis^20,21^. Postmenopausal women are of particular interest as they have a high prevalence of vitamin D deficiency^22^. In that sense, it would be useful to develop a simple new device that raises blood vitamin D levels for postmenopausal female patients with osteoporosis.

Several mechanisms have been speculated to underlie the development of postmenopausal osteoporosis. During menopause, estrogen deficiency impairs the normal bone remodeling cycle by increasing osteoclast resorption activity without increased osteoblast activity. Therefore, the amount of resorbed bone is greater than the amount of bone produced, leading to loss of the total bone mass^23^. In the present study, it could not be shown that the elevated activity of osteoclasts in ovariectomized mice was suppressed by the upregulation of serum vitamin D with UV irradiation (data not shown) probably due to the minimally significant effects of irradiation on the cortical thickness. The indirect and direct effects of estrogen are thought to cause postmenopausal osteoporosis. The production of inflammatory cytokines such as interleukin (IL) -1, IL-6, IL-7, tumor necrosis factor (TNF) -α, granulocyte macrophage colony stimulating factor (GM-CSF) by immune cells is stimulated^24^. Analysis of how UV irradiation affects the inflammatory environment in menopausal estrogen deficiency should be a topic for the future.

An association between vitamin D and sarcopenia has been reported^25,26^. In the present study, UV irradiation did not increase muscle strength which was different from the results of irradiation experiments on SAMP6, aging-promoting mice^14^. An appreciable effect of UV irradiation on muscle strengthening may not be expected for patients with menopausal osteoporosis.

There are several limitations in the present study. First, since the illuminance and dose were set with few side effects, no significant effects of UV irradiation were observed on bone morphology or strength under the conditions of the present study. Much longer duration of UV irradiation may provide greater efficacy for them. Another reason may be that the effects of Vit. D production with UV irradiation may be less in patients with postmenopausal osteoporosis than those with the aged type of osteoporosis. Second, different or larger animal ovariectomy models may give different results^27^. The effects on the increase in bone mineral density (cortical thickness) were significant, but small, with this probably resulting in the lack of any statistical difference being observed in mRNA levels examined. Third, a sufficient supply of vitamin D may be more effective than UV irradiation.

In conclusion, UV-irradiation using a short-range LED device could increase serum levels of Vit. D, leading to increases of BMD and bone strength. Thus far, the therapeutic approach for osteoporosis is limited to exercise, sunbathing, and drug therapy. Use of a therapeutic device for osteoporosis is a modality with a novel concept for osteoporosis, and has the potential to reduce the associated medical costs. Thus further studies are warranted.

## Materials and methods

### Study design: preliminary and main experiments

All animal procedures for experiments were approved by the Animal Care and Use Committee of Nagoya University (license number; 28106) and carried out according to the National Institutes of Health's Guide to the Management and Use of Laboratory Animals. All experiments were performed painlessly under pentobarbital sodium or isoflurane anesthesia and every effort was made to minimize mouse distress. All methods are reported in accordance with ARRIVE guidelines. This study consisted of two stages of experiments. First, we investigated the vitamin D and bone metabolism in ovariectomized mice, a model of postmenopausal osteoporosis, without irradiation as a preliminary experiment. Based on the results of this preliminary treatment, we determined the timing of UV-LED irradiation. Under the determined conditions, UV-LED was irradiated to ovariectomized mice to investigate the effects on vitamin D and bone metabolism, bone mineral density and strength, in addition to the muscle volume and strength as the main experiment.

### Preliminary experiments

#### Mice and diet

Inbred C57BL/6 female mice were obtained from Japan SLC, Inc. (Hamamatsu, Japan). The mice were shielded from normal fluorescent UVB in a 12 h light–dark cycle, and kept at a temperature of 25° C. Mice were fed a standard wheat-based diet until the age of 12 weeks. To make an experimental 25 (OH) D starved mouse group, Mice were fed AIN93G as a vitamin D-containing diet and AIN93GA-2 (Oriental Yeast Co., Ltd., Tokyo, Japan) as a vitamin D-deficient diet until 24 weeks of age when the study protocol was completed^28^. AIN93G contains 1000 IU / kg of vitamin D, 0.50% calcium and 7.00% total fat, whereas AIN93GA-2 contains 0 IU / kg, 0.50% and 7.00%, respectively. To create the postmenopausal osteoporosis model, we ovariectomized mice at 16 weeks of age as described in the previous reports^27,29^. Finally, the mice were randomly divided into four groups (n = 6): (1) Vit.D+ OVX− as normal control; (2) Vit.D+ OVX+ ; (3) Vit.D− OVX− ; (4) Vit.D− OVX+ .

#### Ovariectomy or sham surgery

16-week-old mice were randomly assigned to OVX or sham surgery group, anesthetized with intraperitoneal pentobarbital, and operated on. In short, OVX was performed by a bilateral dorsoventral approach as previously reported^30^. Each ovary was cauterized and resected at the tip of the uterine horn. The sham surgery also made an incision to expose the ovaries, but did not remove the tissues. After ovarian and sham surgery, mice were randomly divided into groups based on experimental design.

#### Serum metabolites

Levels of serum 25(OH)D and 1,25(OH)_2_D were determined at 12 weeks of age (pre-vitamin D diet), 15 weeks (pre-OVX/sham), and 20, 24 weeks (4, 8 weeks after OVX/sham, respectively). The levels were determined with radioimmunoassay kits (SRL, Tokyo, Japan) according to the manufacturer's instruction. Blood samples were taken from the orbital plexus and used for measurement for six mice in each group, and stored at − 20 ℃ until quantification. Levels of the vitamin D were classified as follows: deficient, 25(OH)D < 25 nmol/L or sufficient, 25(OH)D > 90 nmol/L, as described previously^31^.

#### Analyses with micro-computed tomography (CT)

The distal femoral metaphysis was used to assess the effect of ovariectomy on mouse trabecular and cortical microarchitecture. Analysis by micro-CT scan of the metaphysis of the right distal femur was performed every 4 weeks from 12 to 24 weeks (pre- to 8 weeks after OVX) of age for six mice alive in each group, by high resolution micro-CT scanner with a specific software (SkyScan 1176; Bruker, Kontich, Belgium), according to the previous reports^12,32^. Mice were anesthetized with Isoflurane (2.5% flow) and maintained below 2.5% using a nose-cone setup for imaging. Each scan was performed with a power supply voltage of 50 kV, a current of 500 μA, a rotation step of 0.5°, a full rotation of over 180°, and a 0.5 mm aluminum filter for reduced beam hardening. The exposure time was 0.89 s, and the pixel size was 9 µm. The scan also included phantom bones to analyze bone mineral density (250 mg / cm3 and 750 mg / cm3) to standardize grayscale values and maintain consistency between assessments. Three-dimensional (3D) microstructural images were reconstructed using NRecon software (Bruker, Kontich, Belgium), and morphometric parameters were analyzed using the SkyScan CT Analyzer (CTAn) software for trabecular and cortical bone of the femur. To determine cancellous bone morphometry parameters, the volume of interest (VOI) from 0.17 mm from the growth plate of the femur towards the diaphysis (2 mm high), including the trabecular and medullary cavity. To determine cortical bone morphometry parameters, the VOI started at the proximal end of the trabecular bone, and set up to 2 mm towards the central shaft (height 2 mm), targeting only the cortical shell. Bone parameters (bone volume fraction [BV (bone volume) /TV (trabecular volume), %], trabecular thickness [Tb.Th, μm], number [Tb.N, 1/mm], spacing [Tb.Sp, mm], bone mineral density [BMD, mg/cm^3^], and cortical thickness [Ct.Th, mm]) were calculated based on guidelines for analyzing bone microstructure in rodents with micro-CT^33^.

### Main experiments

#### Ovariectomy-induced osteoporotic mice and treatment groups

Thirty-two C57BL/6 female mice were obtained from Japan SLC, Inc. (Hamamatsu, Japan). They were fed either a vitamin D containing or deficient diet from 12 weeks of age, OVX were performed in all mice at 16 weeks of age, and irradiated with UV from 24 to 48 weeks of age based on the results of the preliminary experiment. At 24 weeks of age before irradiation, 32 mice were divided into 4 groups: oral vitamin D-repletion without UV irradiation (Vit.D+ UV−) as a control, oral vitamin D-repletion with UV irradiation (Vit.D+ UV +), oral vitamin D-deficiency without UV irradiation (Vit.D− UV−), and oral vitamin D-deficiency with UV irradiation (Vit.D− UV +). Each group comprised 8 mice. At the age of 48 weeks, the mice were victimized, and specimens were collected, and subjected to RT-PCR analysis, mechanical tests, and histological assays.

#### UV irradiation

In collaboration with Dr. Hiroshi Amano of our hospital, a UV lamp equipped with a surface mount device of an LED system developed by Nikkiso Co., Ltd. (Tokyo, Japan) was used as a UV source. We adjusted to a wavelength of 316 nm of the LED module, because 316 nm, which is in the UVA wavelength range, was already determined to provide Vitamin D efficiently, and confirmed to be less harmful in our previous work^14^. As previously reported, a 2 × 4 cm a dorsal part of the skin was cleanly shaved as the area to be irradiated^34^. Mice were irradiated in a transparent acrylic box with a bottom area of 4 × 6 cm. The lamp was placed 10 cm above the back of the mouse. The irradiance of the dorsal area inside the box by the LED module was calculated using a UV radiometer USR-45DA-10 (Ushio Inc., Tokyo, Japan). The reflectance coefficient in the box was calculated to be 1.77. UV irradiation dose was adjusted to 1000 J / m2 twice a week based on the determination in the previous study^14^.

#### Serum metabolites

Levels of serum 25(OH)D were determined at 15 weeks of age (pre-OVX/sham), 22 weeks (pre-UV irradiation), and 40, 48 weeks (16, 24 weeks’ UV irradiation), and levels of serum 1,25(OH)_2_D at 48 weeks of age. Serum inorganic phosphorus (IP) and calcium (Ca) concentrations were measured immediately after blood collection with standard colorimetric methods using a DryChem (FujiFilm, Tokyo, Japan). Levels of serum 1–84 parathyroid hormone (PTH) were measured using a sandwich ELISA kit (Immutopics, San Clemente, USA). Sera were stored at − 80℃, and subjected to the measurement for PTH.

#### Analyses using micro-computed tomography (CT)

In the same way as in the preliminary experiment, we evaluated the effect of UV irradiation on trabecular and cortical microarchitectures in ovariectomized mice. The right distal femur metaphysis was scanned with micro-CT at 15 weeks of age (pre-OVX/sham), 22 weeks (pre-UV irradiation), and 29, 36, 42, 48 weeks (5, 12, 18, 24 weeks’ UV irradiation) for eight mice alive in each group, Bone parameters measured in the preliminary experiment were also measured in the main one.

#### Mechanical test

The mechanical strength of the right femur was measured by a three-point bending test using a mechanical strength analyzer (MZ500D; Maruto, Tokyo, Japan). Four mice were analyzed in each group. The central diaphysis of the femur was placed on two supports located 6 mm apart on the test device. At the midpoint between the two supports, a three-point bending test load was applied in the anteroposterior direction. Load–displacement curves were recorded at crosshead velocities of 2.0 mm / s. Mechanical parameters [ultimate load (N), stiffness (N/mm), displacement of fracture (mm), and work to failure (N*mm)] were measured with CTR win. Ver. 1.05 software (System Supply, Nagano, Japan).

#### Real-time RT-PCR analysis

To evaluate the effects of UV irradiation on the control of the metabolism of vitamin D (25(OH)D and 1,25(OH)2D), mRNA expression levels of involved enzymes, which mediate the vitamin D metabolic pathway, were analyzed. We also evaluated expression levels of various bone turnover markers to analyze the effects of UV irradiation on the control of bone metabolism. Liver samples were obtained at 48 weeks of age and used for real time RT-PCR to calculate mRNA levels of vitamin D 25-hydroxylase (*Cyp27a1*). Kidney samples obtained at 48 weeks of age were subjected to the analysis of mRNA levels of 25 hydroxyvitamin D-1-alpha hydroxylase (*Cyp27b1*) and 1,25-dihydroxyvitamin D 24-hydroxylase (*Cyp24a1*). Tibia samples at 48 weeks of age were obtained to analyze mRNA levels of alkaline phosphatase (*ALP*), Osteocalcin, Runt-related transcription factor 2 *(Runx2*), Osterix, receptor activator of NFκB ligand (*RANKL*), nuclear factor of activated T cells (*NFATc1*) and nuclear factor kappa B (*NFκB*). RNA was extracted from liver, kidney, and tibia of each mouse using the RNeasy Mini Kit (Qiagen, Hilden, Germany) under the instruction of the supplier’s description. After reverse transcription, the cDNA was used for real-time RT-PCR using a LightCycler 480 (Roche Diagnostics, Mannheim, Germany), with 480 SYBR Green I Master (Roche Diagnostics, Mannheim, Germany), with 0.5 μM of the specific sense and antisense primers. Amplification protocol was as follows; denaturation of the template cDNA for 10 min at 95 °C, 45 cycles of a denaturation step for 10 s at 95 °C and an annealing step for 10 s at 60 °C and an extension step for 10 s at 72 °C. All PCRs contained a negative control that did not include a cDNA template. To confirm the specificity of the amplified products, the PCR products were subjected to melting curve analysis with LightCycler 480 and 2% agarose / TAE gel electrophoresis to measure Tm and amplicon sizes, respectively. To allow relative quantification after PCR, the LightCycler 480 software (Roche Diagnostics, Mannheim, Germany) was diluted from the specified gradient to calculate real-time efficiency. The levels of mRNA in the sample were calculated as relative values normalized at the level of glyceraldehyde-3-phosphate dehydrogenase (Gapdh). The primer pairs for *Gapdh, Cyp27a1, Cyp27b1, Cyp24a1, ALP*, *Osteocalcin, Runx2*, *Osterix, RANKL*, *NFATc1,* and *NFκB* were designed according to previous reports^35,36^.

#### Bone histology

To assess bone formation in non-decalcified bone, the resected left bone was analyzed by Villanueva Goldner staining. A 48-week-old sample was fixed with 70% ethanol for 3 days, dehydrated stepwise with ethanol, and embedded in glycol methacrylate without decalcification (Aichi Pathologic Laboratory, Aichi, Japan). A 30 µm coronary section was made from the embedded tissues, stained with Villanueva Goldner, and analyzed under a light microscope.

#### Grip strength measurement

Grip strength of the forefoot of mice was tested in each group at 48 weeks of age using a grip strength meter (Columbus Instruments, OH, USA), and recorded in Newtons (N). In brief, the tail of the mouse was held by the examiner's finger, and the forearm of the mouse held the handle. The examiners pulled the mouse body parallel to the floor with their fingers. Three sets of five tests were performed on each mouse, with short breaks between sets. Mean values of grip strength were determined.

### Statistics

Results are expressed as mean ± standard deviation (SD). The Kruskal–Wallis test and the Mann–Whitney U test were applied to compare the results. SPSS statistics version 24 (IBM Corp. Armonk, NY) were used for all statistical analyses. *p* < 0.05 was considered as statistically significant.
